# An Experimental Study in Laser-Assisted Machining of AerMet100 Steel

**DOI:** 10.3390/mi15070926

**Published:** 2024-07-20

**Authors:** Yu Tang, Yugang Zhao, Shuo Meng, Yusheng Zhang, Qilong Fan, Shimin Yang, Guiguan Zhang, Jianbing Meng

**Affiliations:** School of Mechanical Engineering, Shandong University of Technology, Zibo 255049, China; tangyu1232022@126.com (Y.T.); m1369160775@126.com (S.M.); zys19862734240@126.com (Y.Z.); fql0317@126.com (Q.F.); ysmsdut@126.com (S.Y.); zhanggg1006@163.com (G.Z.); jianbingmeng@sdut.edu.cn (J.M.)

**Keywords:** laser-assisted machining (LAM), AerMet100 steel, surface roughness, surface morphology, tool wear

## Abstract

To solve the problems of poor surface quality and low tool life in conventional machining (CM) of AerMet100 steel, an experimental study was conducted in laser-assisted machining (LAM) of AerMet100 steel. The effects of laser power, cutting speed, feed rate, and depth of cut on the surface roughness of AerMet100 steel were studied based on a single-factor experiment. The degree of influence of each factor on the surface roughness was evaluated by analyses of variance and range in the orthogonal experiment, and the combination of process parameters for the optimal surface roughness was obtained. The order of influence was as follows: laser power > cutting speed > depth of cut > feed rate; the optimal combination of process parameters was laser power 200 W, cutting speed 56.5 m/min, feed rate 0.018 mm/rev, and depth of cut 0.3 mm. Compared to CM, the surface morphology of the workpiece under the optimization of LAM was relatively smooth and flat, the surface roughness Ra was 0.402 μm, which was reduced by 62.11%, the flank wear was reduced from 208.69 μm to 52.17 μm, there were no tipping or notches, and the tool life was significantly improved. The study shows that the LAM of AerMet100 steel has obvious advantages in improving surface quality and reducing tool wear.

## 1. Introduction

As an ultra-high strength alloy, AerMet100 steel is widely used in aerospace, military, energy, and other fields because of its outstanding mechanical properties such as ultra-high tensile strength, fracture toughness, excellent resistance to stress corrosion cracking, and fatigue [1,2,3,4]. However, AerMet100 steel belongs to the typical difficult-to-machine materials; its high strength, high toughness, and low thermal conductivity lead to poor surface quality, low tool life, and difficult chip breaking in conventional machining (CM). Laser-assisted machining (LAM) is an emerging composite-machining technology applied to difficult-to-machine materials; it uses the thermal effect of the laser beam to soften the workpiece material in the cutting area and reduce the yield strength of the material so as to solve the problems existing in CM and achieve high-quality machining.

AerMet100 steel is an ultra-high strength martensitic steel (yield strength exceeds 1400 Mpa), because its strength comes from the secondary hardening produced by tempering of low-carbon high-alloy martensitic below 550 °C [5]; one way of machining is to first rough the blank material with low hardness, obtain the approximate shape of the workpiece, then achieve the required hardness through heat treatment, and finally, fine machine by grinding [6]. The problem with this method is that the heat-treatment process has a greater effect on the overall dimensional control, and the subsequent grinding process makes it difficult to correct any workpiece deformation caused by heat treatment [7,8]. At the same time, the material removal rate of grinding is low, the machining process is costly and time-consuming, and the grinding heat also has an adverse effect on the machining quality and fatigue resistance of the parts [9,10]. Since the mid-1980s, with the development of superhard cutting tools, hard machining has gradually become a conventional processing method. Hard machining is the machining of parts with a hardness of 45 HRC or more; compared to other methods, hard machining involves the heat treatment of the parts before machining [11]. The advantage of this method is that it avoids the problems caused by heat treatment during machining and does not require multiple clamping, which can ensure the machining accuracy requirements [12]. However, there are some limitations in hard machining: it requires high cutting forces, high rigidity requirements for the machining system, high tool costs as well as low tool life, and poor machined surface quality [13]. In order to overcome the shortcomings of the above-mentioned processing methods and meet the increasing demand for high-strength materials, heat-assisted cutting technology was proposed. Based on CM, the technology introduces an external heat source to soften the material in the local area, making the material removal easier. The commonly used external heat sources include plasma [14], gas flame [15], laser [16], etc. Among them, the laser has the advantages of high energy density, precise control of the heating process, wide applicability, and easy control of the heat-affected zone [17]. Therefore, LAM has become a research hotspot in the field of special machining. The existing research shows that LAM has been applied to a variety of high-strength alloys. Anderson et al. carried out LAM of Inconel 718; the results demonstrated that the specific cutting energy and surface roughness were reduced greatly, and the tool life was also improved under the material-removal temperature, which reached 620 °C [18]. Xavierarockiaraj et al. determined the parameter range of LAM of SKD11 tool steel by laser heating experiments and analyzed the influence of cutting speed and feed rate on cutting force, surface roughness, and tool wear in a wide range. The results showed that cutting force and surface roughness were reduced by 40% and 50%, respectively, compared to that of CM [19]. Panjehpour et al. used pulsed laser-assisted machining of AISI52100 bearing steel to study the microstructure and microhardness of CM and LAM of the workpiece at different laser powers. In this research, the subsurface microstructure and microhardness of the workpiece did not change significantly [20]. Ding et al. reported the surface roughness, size control, and residual stress of LAM of AISI4130-hardened steel. It was shown that the surface roughness of the machined workpieces was good, and the diameter of the workpiece produced by LAM and CM was consistent, which could achieve accurate dimensional control. Compared to CM, the axial residual compressive stress increased by about 150 MPa, and the circumferential stress fluctuation decreased [21]. Dumitrescu et al. indicated that for AISID2 tool steel, the application of LAM could reduce the cutting force, avoid catastrophic failure of the tool, and inhibit the formation of machining chatter and saw-tooth chip [22]. Khatir et al. studied the effect of process parameters on surface roughness during LAM of AISI4340 and found that the appropriate combination of process parameters could effectively avoid thermal damage and maximize the advantages of LAM [23]. Venkatesan et al. used the Taguchi orthogonal array method to conduct experimental research on the LAM of Inconel 718. It was concluded that the cutting speed and laser power had a large degree of influence on the cutting force, followed by the feed rate; the optimum level of process parameters was obtained by signal-to-noise ratio analysis and carried-out experiments, at which time the cutting force reduced by 60% [24].

The excellent research results of the predecessors have fully demonstrated that in the machining of high-strength alloys, compared to the problems of poor surface quality and serious tool wear in CM, LAM has absolute advantages in improving the surface integrity of the workpiece and reducing the tool-wear rate. Therefore, this paper explored the LAM of AerMet100 steel, analyzed the law of influence of process parameters on surface quality, and systematically studied the optimal combination of LAM parameters to obtain higher surface quality and smaller tool wear.

In this study, the effects of laser power, cutting speed, feed rate, and depth of cut on surface roughness were, firstly, investigated by a single-factor experiment. Then, the degree of influence of each factor on the surface roughness was evaluated by an analysis of variance and of range in the orthogonal experiment, and the combination of process parameters for the optimal surface roughness was obtained. Finally, the surface morphology and tool wear were compared to CM.

## 2. Experimental

### 2.1. Experimental Material and Equipment

AerMet100 steel (Shandong Hastelloy Co., Ltd., Liaocheng, China), with a length of 100 mm and a diameter of 20 mm, was used as experimental material. The main chemical composition and thermal physical parameters of AerMet100 steel are listed in Table 1 and Table 2, respectively. Table 1 shows that the total content of alloying elements in AerMet100 steel is about 29%, in which Co, Ni, and Cr elements are beneficial to improve the strength, rust, and corrosion resistance of the material [25]. Table 2 shows that the lower thermal conductivity is an important factor that contributes to its poor cutting performance.

**Figure 1:**
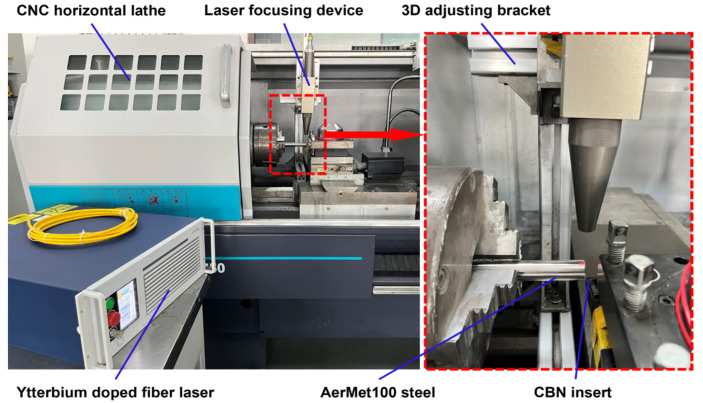
The laser-assisted machining experimental system.

The experimental platform of this study consisted of a turning machining system and a laser-assisted heating system, as shown in Figure 1. The turning machining system was composed of a CNC horizontal lathe (CKD6136i, Dalian Machine Tools Group, Dalian, China) with a FANUC system. The maximum speed of the machine tool spindle was 3000 r/min, and the repeatability of the positioning accuracy was 8 μm. LAM of AerMet100 steel was carried out using a CBN insert (CNGA120408 FBS7000) mounted on a composite compression-type tool holder (MCLN2020K12). The CBN insert has high heat resistance and chemical stability, high hardness at high temperatures, low friction coefficient, and high machining accuracy, which is suitable for application in LAM [26]. The laser-assisted heating system consisted of a ytterbium-doped fiber laser (YLR-150/1500-QCW-MM-AC, IPG Photonics Co., Oxford, MI, USA) and a laser focusing device. The laser has a wavelength of 1070 nm and a maximum average power of 250 W in continuous mode. The laser source was transmitted through the fiber-optic cable and sent to the turning machining system through the laser focusing device. Finally, the laser beam was irradiated vertically on the surface of the workpiece. The laser focusing device was installed on the three-dimensional adjusting bracket attached to the moving guide rail base of the lathe; it can change the position of the laser beam and the spot size.

### 2.2. Experimental Principle

**Figure 2:**
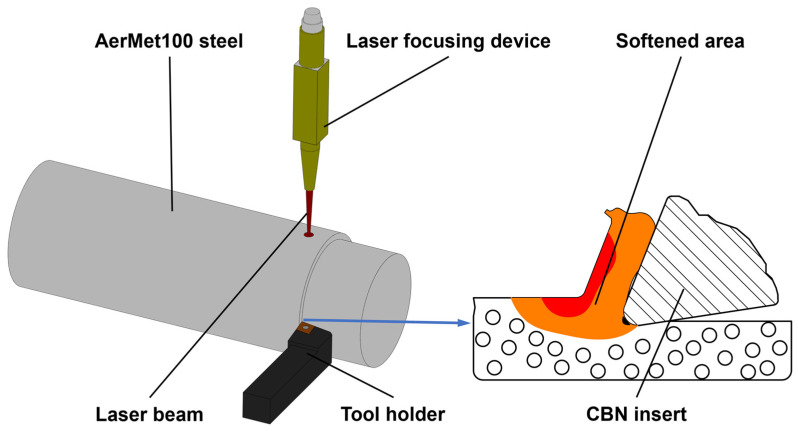
The principle of LAM of AerMet100 steel.

The principle of LAM of AerMet100 steel is shown in Figure 2. This technology combined laser technology with conventional turning machining; the high-energy laser beam was focused vertically on the AerMet100 steel bar, and the laser beam was always located in front of the tool and maintained synchronous feed with the tool. The hardness and yield strength of the workpiece material in the area to be cut were reduced under the thermal effect of the laser, and the cutting performance was improved. Zeng tested the strength of AerMet100 steel at different temperatures using a thermal simulation machine. The results showed that the strength of AerMet100 steel gradually decreased with the increase in temperature, and when the temperature was 600 °C, the ultimate compressive strength and yield strength decreased by about 57.1% and 46.9%, respectively [27]. The hardness of the material was reduced to 40 HRC, and the material removal became easy. LAM has significant advantages over conventional turning in terms of reducing the cutting force and specific cutting energy, reducing the friction between the tool and the machined surface, prolonging the tool life, and improving surface quality and machining efficiency [28,29].

### 2.3. Experimental Design

There are two main factors that affect the machining quality of AerMet100 steel for LAM. One is the turning machining system, in which the cutting speed, feed rate, and depth of cut are important parameters, and the other is the laser-assisted heating system, in which the laser power is an important laser parameter that directly affects the surface temperature of the heated workpiece, thus influencing the cutting performance. Therefore, the four process parameters of cutting speed, feed rate, depth of cut, and laser power were selected as experimental factors.

A single-factor experiment and an orthogonal experiment were carried out. The influence of four process parameters on the machined surface roughness of AerMet100 steel was studied by single-factor experiment, and then an L_16_ (4^4^) standard orthogonal array (OA) was constructed for the orthogonal experiment. The degree of influence of different process parameters on surface roughness and the optimal combination of process parameters were analyzed. The experimental parameters are shown in Table 3 and Table 4. According to the results of previous studies, combined with simulation and exploratory experiments, the laser spot diameter was determined to be 1.1 mm, and the distance between the center of the laser spot and the tip of the tool was set at 1 mm in the axial direction; the preheating time was 15 s, which ensured that the workpiece reached a good preheating effect before cutting. The CBN insert was replaced with a new one for each experiment. The machined workpieces were ultrasonically cleaned with anhydrous ethanol, and the ultra-depth 3D microscope (DSX1000, OLYMPUS, Tokyo, Japan) was used to measure the surface roughness. The surface roughness was measured according to the GB/T 1031-1995 standard. Five times the sampling length was set as the evaluation length during the measurement. The sampling length was 0.8 mm, and the cut-off length was 0.8 mm. The surface roughness Ra was measured at three different positions on the feed direction of the machined surface, and the average value was taken as the final measurement result.

## 3. Results and Discussions

### 3.1. Single-Factor Experiment Results and Analysis

#### 3.1.1. Effect of Laser Power on Surface Roughness

**Figure 3:**
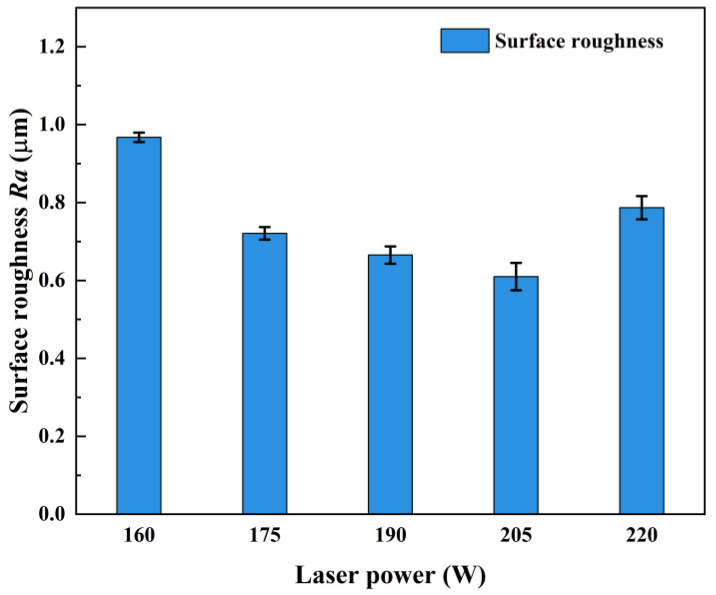
Effect of laser power on surface roughness.

The measured results of the machined surface roughness of AerMet100 steel at different laser powers are shown in Table 5, and the trend of surface roughness with laser power is shown in Figure 3. With the increase of the laser power, the surface roughness decreased first and then increased, and when the laser power was 205 W, the minimum surface roughness was 0.61 μm. When the laser power was less than 205 W, the increase of laser power made the temperature of the cutting area of the workpiece gradually increase, the strength and flow stress of the workpiece material gradually decrease, the cutting force required for material removal decrease, and the load on the tool and the extrusion and friction between the tool and the workpiece reduce. Therefore, the flank wear of the tool was reduced, and the surface roughness was improved. When the laser power was greater than 205 W, the temperature of the tool-workpiece interface rose further, and the flank wear was aggravated; at the same time, the excessively high temperature made the workpiece material easy to bond and accumulate on the cutting edge, which had an adverse effect on the surface quality of the machined surface. In addition, the high temperature led to a further increase in the softening degree of the material, and the machined surface underwent a large plastic flow under the extrusion of the tool, so the surface roughness increased.

#### 3.1.2. Effect of Cutting Speed on Surface Roughness

**Figure 4:**
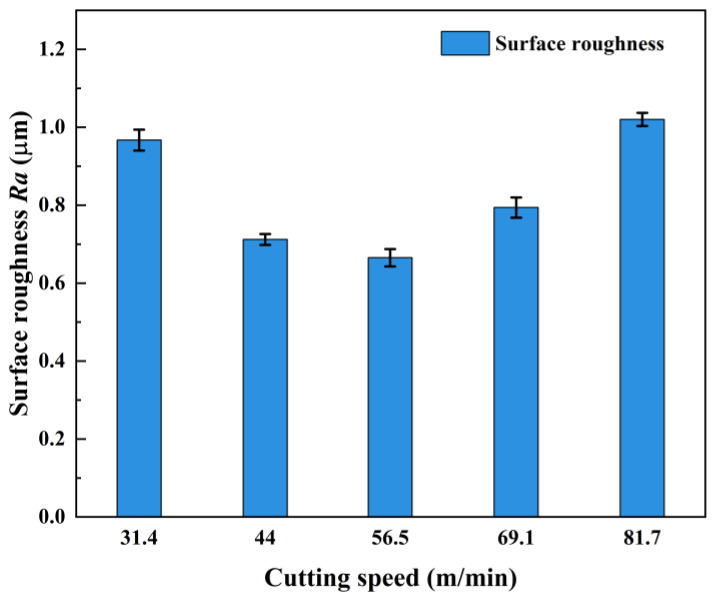
Effect of cutting speed on surface roughness.

The measured results of the machined surface roughness of AerMet100 steel at different cutting speeds are shown in Table 6, and the trend of surface roughness with cutting speed is shown in Figure 4. With the increase of the cutting speed, the surface roughness decreased first and then increased, and when the cutting speed was 56.5 m/min, the minimum surface roughness was 0.665 μm. When the cutting speed was too low, the energy distribution was not uniform. In the same heating area, the interaction time between the workpiece surface and the laser beam was too long, and the softening degree of the material was too high. When the material was removed, it was prone to uneven plastic deformation, and the surface roughness increased. When the cutting speed was too high, due to the total energy generated by laser irradiation during machining remaining unchanged, the energy loss through heat convection and heat transfer in the laser irradiation area at high speed increased, and the high cutting speed made the heating time of the laser beam to the cutting area shorter, which eventually led to a reduction in the temperature of the cutting area, a decrease in the thermal softening effect of the material, an increase in the tearing of the material during the machining, and an increase in the surface roughness. In addition, the excessively high cutting speed also enhanced the extrusion friction between the tool and material, resulting in increased tool wear and decreased surface quality.

#### 3.1.3. Effect of Feed Rate on Surface Roughness

**Figure 5:**
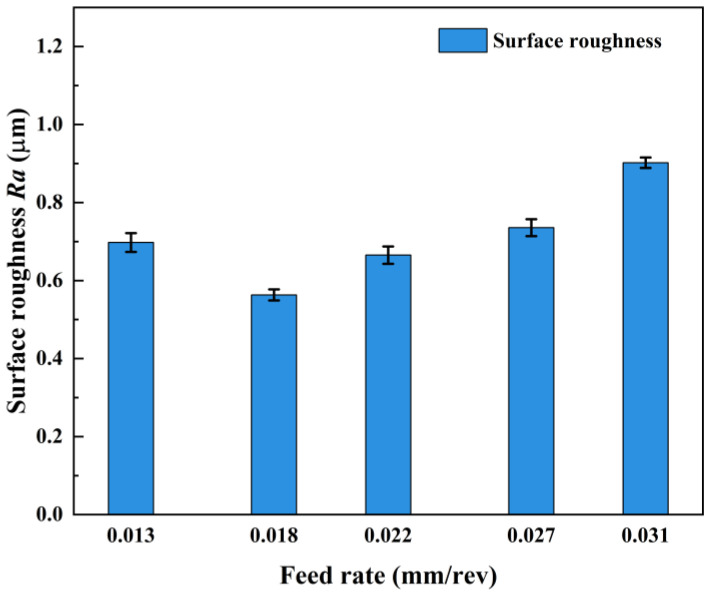
Effect of feed rate on surface roughness.

The measured results of the machined-surface roughness of AerMet100 steel at different feed rates are shown in Table 7, and the trend of surface roughness with feed rate is shown in Figure 5. With the increase in feed rate, the surface roughness decreased first and then increased gradually. When the feed rate was 0.018 mm/rev, the minimum surface roughness was 0.563 μm. When the feed rate is 0.013 mm/rev, the smaller feed rate caused the laser to stay in the cutting area for too long, and the material absorbed more heat to make the temperature too high. On the one hand, the visco-plasticity was enhanced too much, and the excessive fluidity made the material undergo a larger plastic shear deformation under the action of the tool’s extrusion; on the other hand, during the feeding process, the high temperature caused the bond on the cutting edge of the tool to gradually accumulate, which easily caused damage to the machined surface and increased the surface roughness. When the feed rate increased to 0.018 mm/rev, the plastic effect of the material caused by thermal deposition was more suitable for machining, so as to obtain good surface roughness. When the feed rate increased from 0.018 mm/rev to 0.031 mm/rev, the irradiation time per unit length of the laser in the axial direction of the workpiece gradually decreased, the temperature of the cutting area gradually decreased, the softening degree of the material decreased, the friction and plowing effect in the cutting process became more and more significant, the tool wear rate rose, and the surface roughness increased. In addition, the increase in feed rate increased the volume of material to be removed per unit of time, the energy required in the cutting process rose, the cutting force and tool vibration rose, and the surface roughness increased.

#### 3.1.4. Effect of Depth of Cut on Surface Roughness

**Figure 6:**
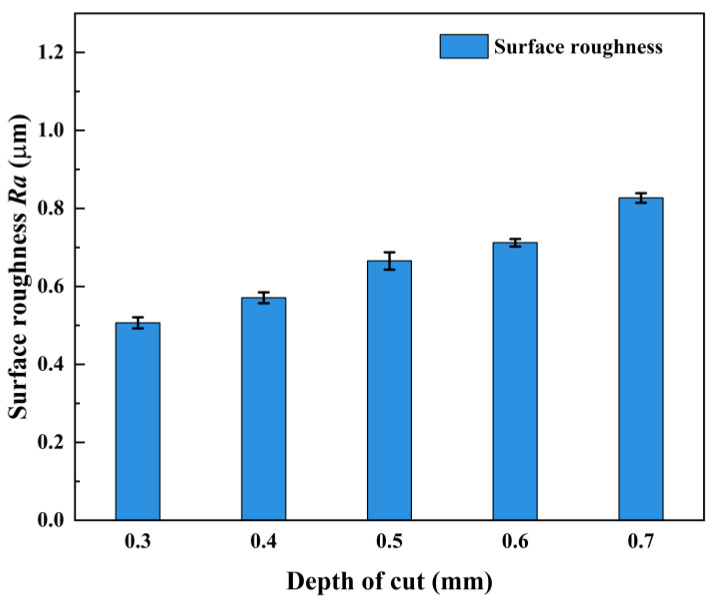
Effect of depth of cut on surface roughness.

The measured results of the machined surface roughness of AerMet100 steel at different depths of cut are shown in Table 8, and the trend of surface roughness with depth of cut is shown in Figure 6. With the increase in the depth of the cut, the surface roughness increased gradually. When the depth of the cut was 0.3 mm, the minimum surface roughness was 0.506 μm. As the laser power and heating time remained unchanged, the degree of thermal penetration of the material was constant. The increase in the depth of the cut led to a decrease in the temperature in the shear zone of the material, which weakened the plastic deformation ability of the material—the mechanical load of the tool increased, the tool wear increased, and the surface roughness increased. If the depth of the cut was too high, the thickness of the softened layer under the action of the laser beam was small, and the deeper material was almost no longer affected by the thermal effect of the laser beam [30]. At that time, the cutting state was close to hard machining, the cutting force and tool vibration increased, the cutting process became unstable, the scratches, burrs, and other defects on the machined surface were too many, and the surface quality decreased sharply.

### 3.2. Orthogonal Experiment Results and Analysis

The surface roughness results of the orthogonal experiment are shown in Table 9, and the machined-surface roughness values of AerMet100 steel were in the range of 0.436–0.820 μm. Through analysis of variance and analysis of range, the significant degree and order of priority of the influence of laser power, cutting speed, feed rate, and depth of cut on surface roughness were explored, and the process parameters were reasonably optimized and matched to obtain the combination of process parameters for the optimal surface roughness.

#### 3.2.1. Analysis of Variance

The results of the analysis of variance for surface roughness are shown in Table 10. By comparing the F-value, the degree of influence of each process parameter on the surface roughness was judged. The F-values corresponding to laser power, cutting speed, feed rate, and depth of cut were 7.02, 1.02, 0.86, and 0.46, respectively. This indicates that laser power has the highest influence on surface roughness, followed by cutting speed and depth of cut, and the feed rate has the lowest influence on surface roughness within the selected experimental parameters. Among the factors affecting surface roughness, laser power dominates. The variation of the laser power over a wide range affects the softening degree of the cutting area of the material, rendering a large difference in the hardness and strength of the material between the different experimental groups, which leads to a higher degree of influence of the laser power on the surface roughness.

#### 3.2.2. Analysis of Range

**Figure 7:**
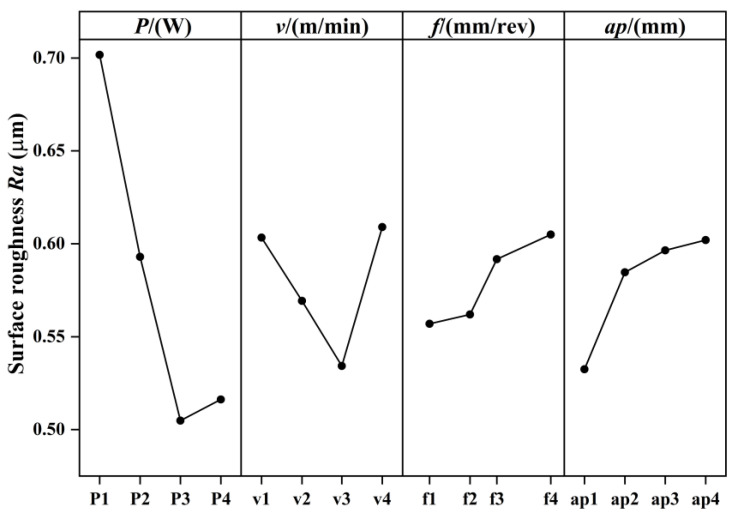
Main effect of mean on surface roughness.

The results of the analysis of range for surface roughness are shown in Table 11. The smaller the K_i_ (i = 1, 2, 3, 4), the smaller the surface roughness value at this level. The R-value reflects the fluctuation of each factor level, and the larger the R-value, the greater the influence of this factor on surface roughness. The R-values corresponding to laser power, cutting speed, feed rate, and depth of cut are 0.1969, 0.0747, 0.0480, and 0.0695, respectively. Therefore, the order of influence of the four factors on surface roughness is laser power > cutting speed > depth of cut > feed rate. The changing trend of K_i_ of each factor at different levels is shown in Figure 7, and through the main effect of mean analysis, it can be seen that the combination of process parameters corresponding to the minimum surface roughness is laser power 200 W, cutting speed 56.5 m/min, feed rate 0.018 mm/rev, and depth of cut 0.3 mm. This combination of process parameters was used for the LAM of AerMet100 steel; the machined surface roughness was 0.402 μm, which is lower than the surface roughness obtained by experiment No. 15 in the orthogonal experiment.

### 3.3. Comparison with Conventional Machining

In order to study the effect of laser-assisted machining optimization, we carried out a comparative experiment of LAM and CM and observed and compared the surface morphology and tool wear of the two different processing methods.

#### 3.3.1. Surface Morphology

**Figure 8:**
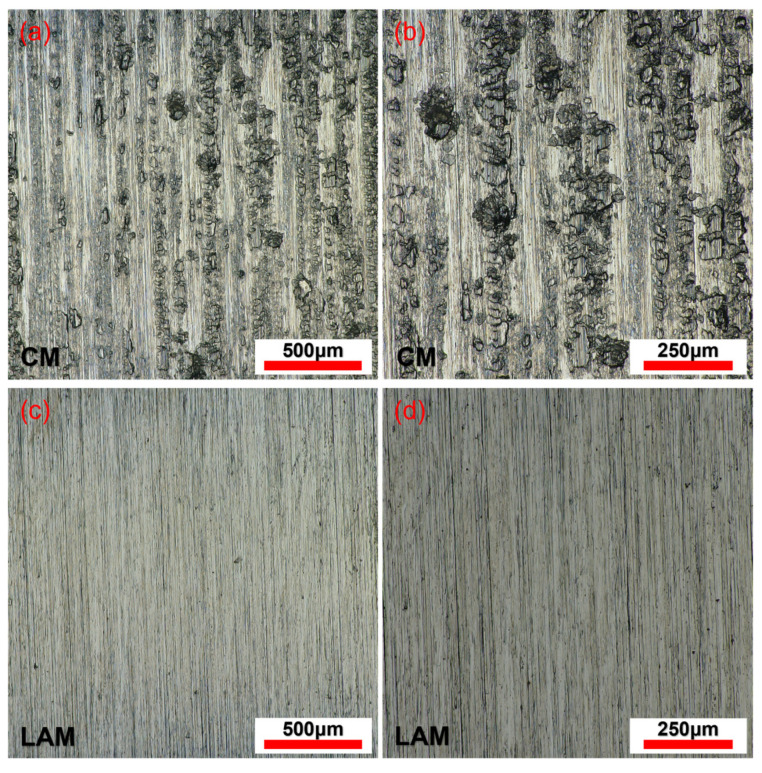
Surface morphology of the machined workpieces at the magnifications of (**a**,**c**) 144× and (**b**,**d**) 258×.

The surface morphology of the workpiece after CM and LAM is shown in Figure 8. Figure 8a,b shows the surface morphology of the workpiece obtained by CM, and it shows that the machined surface was seriously damaged, and a large number of tool scratches and pits were distributed, accompanied by bonding defects caused by material accumulation. The reasons for this phenomenon are that the hardness and strength of AerMet100 steel are high without a heat source in CM, the friction and extrusion between the tool and the machined surface are severe during cutting, and the material was seriously torn, resulting in obvious scratches and pits in the tool. At the same time, the tool wear was fast, and the surface quality deteriorated rapidly. In addition, the signs of tool scratching also indicate that the tool vibrated violently during cutting, and the cutting process was extremely unstable. Figure 8c,d shows the surface morphology of the workpiece obtained by LAM; it shows that the surface was relatively smooth and flat, the machining marks were light, and the overall morphology had no obvious defects. There were only a few micro-pits and fine tool marks. The overall surface quality was significantly improved compared to that of CM. This is because the irradiation of a high-energy laser beam makes the temperature of the area to be cut rise rapidly, the hardness and strength of the material reduce, the material is easy to remove, and the mechanical load borne by the tool as well as the friction between the workpiece and tool reduce. Compared to CM, the degree of tool wear greatly decreases, the tool vibration reduces during the cutting process, the cutting is more stable, and the surface quality is improved.

**Figure 9:**
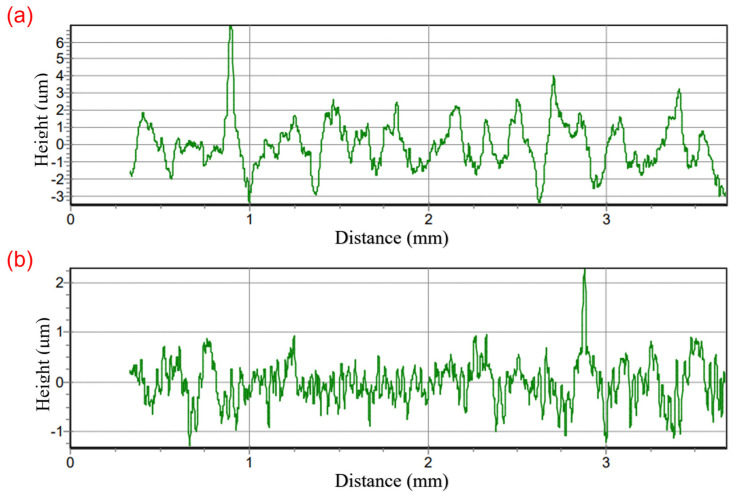
Surface profile curves of the machined workpieces. (**a**) CM; (**b**) LAM.

Figure 9 shows the surface profile curves of the machined surface in CM and LAM. After measurement, the surface roughness of the workpiece in CM was 1.061 μm, while the surface roughness of the workpiece in LAM was 0.402 μm; the surface roughness value decreased by 62.11%, which proves that LAM can effectively reduce surface roughness and improve surface quality.

#### 3.3.2. Tool Wear

**Figure 10:**
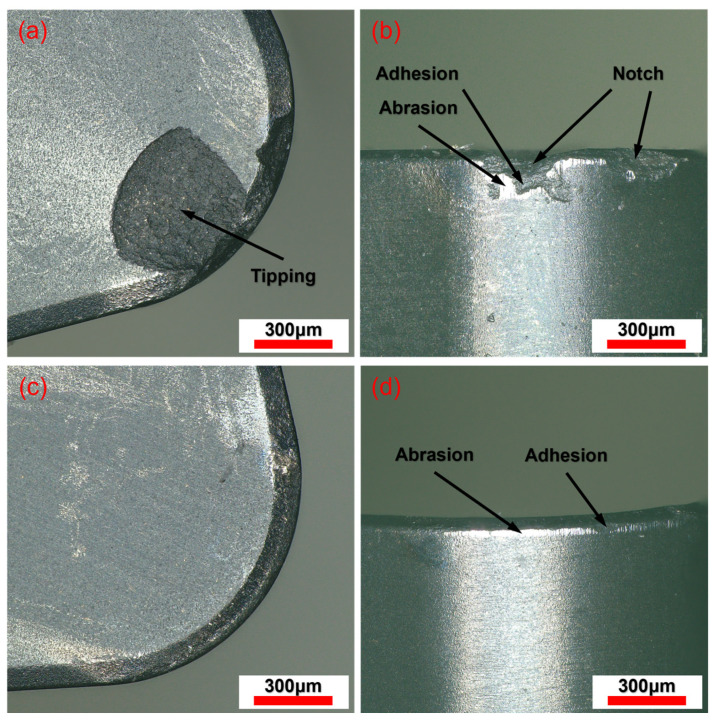
The wear of the rake and flank faces of the tool. (**a**,**b**) CM; (**c**,**d**) LAM.

In turning AerMet100 steel, tool wear is a key factor affecting machining costs and surface quality. In the turning process, tool wear mainly manifests as tool surface wear, cutting edge wear, and chipping, which are caused by the change of cutting force and cutting heat. The wear of the rake and flank faces of the tool under CM and LAM is shown in Figure 10. It shows that the wear mechanism of the tool in CM occurs through tipping at the cutting edge and abrasion and adhesion on the flank face. The wear mechanism of the tool in LAM is mainly characterized by abrasion and adhesion. In the CM process, high strength, hardness, and hard-point particles of the material lead to intense friction between the tool and the workpiece, and the cutting force is large. Figure 10a,b shows that the rake face of the tool causes the tipping, the flank wear is serious, and there are some notches at the cutting edge, which may be due to the stress as the cutting edge reaches the brittle fracture limit of the tool material. In addition, it can also be found that the maximum wear and average wear on the flank face are quite different, and the tool wear is not uniform, which is caused by the large temperature gradient. In the LAM progress, laser heating increases the temperature of the material, reduces the hardness and strength, makes the plastic flow more likely to occur when the material is removed, and reduces the cutting force. At the same time, due to the thermal effect, the abrasiveness of the hard intermetallic phases and abrasive carbides in the AerMet100 steel is reduced, and the friction between the tool-workpiece interface is weakened. Finally, Figure 10c,d shows that the tool wear is only manifested as a slight wear of the cutting edge and the flank face; compared to CM, the rake face has a good morphology and, basically, no wear, and the flank wear is reduced from 208.69 μm to 52.17 μm; the notches at the cutting edge disappear. In addition, due to the increase in material removal temperature, the temperature gradient generated by the tool is reduced, and the flank wear is uniform.

## 4. Conclusions

In this paper, an experimental study of LAM of AerMet100 steel is discussed. The influence of different process parameters on surface roughness was studied by single-factor experiments. The degree of influence of different process parameters on surface roughness and the optimal combination of process parameters were obtained by an orthogonal experimental analysis of variance and an analysis of range. Finally, the surface morphology and tool wear under CM and LAM were compared. The main conclusions are as follows:

1. The effects of laser power, cutting speed, feed rate, and depth of cut on the surface roughness of AerMet100 steel were studied by single-factor experiments. The results show that the surface roughness decreases first and then increases with the increase of laser power, cutting speed, and feed rate, and the surface roughness gradually increases with the increase of depth of cut. When the single-factor levels are 205 W, 56.5 m/min, 0.018 mm/rev, and 0.3 mm, the corresponding minimum surface roughness Ra is 0.61 μm, 0.665 μm, 0.563 μm, and 0.506 μm, respectively.
2. According to the results of the orthogonal experiment, an analysis of variance and an analysis of range were carried out to obtain the degree of influence of four process parameters on surface roughness: laser power > cutting speed > depth of cut > feed rate. The combination of process parameters for the optimal surface roughness are laser power 200 W, cutting speed 56.5 m/min, feed rate 0.018 mm/rev, and depth of cut 0.3 mm, and the corresponding surface roughness Ra is 0.402 μm.
3. The comparison results of surface morphology and tool wear in CM and LAM show that the machined surface of CM has more defects and serious surface damage, and the machined surface of LAM is relatively smooth and flat, and the surface roughness Ra is 0.402 μm, which is reduced by 62.11%. Compared to CM, the flank wear is reduced from 208.69 μm to 52.17 μm, there are no tipping, notches, or other forms of tool failure, and the tool life is significantly improved.

## Figures and Tables

**Table 1:** Main chemical composition of AerMet100 steel.

Element	C	Ni	Cr	Ti	Mo	Co	Fe
Wt. (%)	0.238	11.18	3.00	≤0.015	1.20	13.42	Balance

**Table 2:** Main thermophysical parameters of AerMet100 steel.

Parameter	Unit	Numerical Value
Density	Kg/m^3^	7889
Poisson’s ratio Hardness	- HRC	0.28 54
Tensile strength	Mpa	2000
Specific heat	J/kg·K	412.7
Young’s modulus	MPa	1858
Heat conductivity	W/m^2^·K	19.3

**Table 3:** Single-factor experiment.

Level	Laser Power *P*/(W)	Cutting Speed *v*/(m/min)	Feed Rate *f*/(mm/rev)	Depth of Cut *a*_p_/(mm)
1	160	31.4	0.013	0.3
2	175	44.0	0.018	0.4
3	190	56.5	0.022	0.5
4	205	69.1	0.027	0.6
5	220	81.7	0.031	0.7

**Table 4:** Orthogonal experiment.

Level	Laser Power *P*/(W)	Cutting Speed *v*/(m/min)	Feed Rate *f*/(mm/rev)	Depth of Cut *a*_p_/(mm)
1	180	44.0	0.018	0.3
2	190	50.3	0.021	0.4
3	200	56.5	0.023	0.5
4	210	62.8	0.027	0.6

**Table 5:** Experimental results at different laser powers.

No.	Laser Power *P*/(W)	Cutting Speed *v*/(m/min)	Feed Rate *f*/(mm/rev)	Depth of Cut *a*_p_/(mm)	Surface Roughness *Ra*/(μm)
1	160	56.5	0.022	0.5	0.967
2	175	56.5	0.022	0.5	0.721
3	190	56.5	0.022	0.5	0.665
4	205	56.5	0.022	0.5	0.61
5	220	56.5	0.022	0.5	0.787

**Table 6:** Experimental results at different cutting speeds.

No.	Laser Power *P*/(W)	Cutting Speed *v*/(m/min)	Feed Rate *f*/(mm/rev)	Depth of Cut *a*_p_/(mm)	Surface Roughness *Ra*/(μm)
1	190	31.4	0.022	0.5	0.967
2	190	44.0	0.022	0.5	0.712
3	190	56.5	0.022	0.5	0.665
4	190	69.1	0.022	0.5	0.794
5	190	81.7	0.022	0.5	1.020

**Table 7:** Experimental results at different feed rates.

No.	Laser Power *P*/(W)	Cutting Speed *v*/(m/min)	Feed Rate *f*/(mm/rev)	Depth of Cut *a*_p_/(mm)	Surface Roughness *Ra*/(μm)
1	190	56.5	0.013	0.5	0.698
2	190	56.5	0.018	0.5	0.563
3	190	56.5	0.022	0.5	0.665
4	190	56.5	0.027	0.5	0.736
5	190	56.5	0.031	0.5	0.902

**Table 8:** Experimental results at different depths of cut.

No.	Laser Power *P*/(W)	Cutting Speed *v*/(m/min)	Feed Rate *f*/(mm/rev)	Depth of Cut *a*_p_/(mm)	Surface Roughness *Ra*/(μm)
1	190	56.5	0.022	0.3	0.506
2	190	56.5	0.022	0.4	0.571
3	190	56.5	0.022	0.5	0.665
4	190	56.5	0.022	0.6	0.712
5	190	56.5	0.022	0.7	0.817

**Table 9:** Results of the orthogonal experiment.

No.	Laser Power *P*/(W)	Cutting Speed *v*/(m/min)	Feed Rate *f*/(mm/rev)	Depth of Cut *a*_p_/(mm)	Surface Roughness *Ra*/(μm)
1	180	44.0	0.018	0.3	0.616
2	180	50.3	0.021	0.4	0.671
3	180	56.5	0.023	0.5	0.700
4	180	62.8	0.027	0.6	0.820
5	190	44.0	0.021	0.5	0.657
6	190	50.3	0.018	0.6	0.597
7	190	56.5	0.027	0.3	0.518
8	190	62.8	0.023	0.4	0.600
9	200	44.0	0.023	0.6	0.555
10	200	50.3	0.027	0.5	0.497
11	200	56.5	0.018	0.4	0.483
12	200	62.8	0.021	0.3	0.484
13	210	44.0	0.027	0.4	0.585
14	210	50.3	0.023	0.3	0.512
15	210	56.5	0.021	0.6	0.436
16	210	62.8	0.018	0.5	0.532

**Table 10:** Surface roughness results from analysis of variance.

Source	Degree-of-Freedom (DF)	Sum-of-Squares (SS)	Mean-of-Squares (MS)	F-Value
Laser Power (*P*)	3	0.098857	0.032952	7.02
Cutting speed (*v*)	3	0.014343	0.004781	1.02
Feed rate (*f*)	3	0.006446	0.002149	0.46
Depth of cut (*a*_p_)	3	0.012122	0.004041	0.86
Error (*e*)	3	0.014081	0.004694	
Total	15	0.145849		

**Table 11:** Surface roughness results from analysis of range.

Value	Laser Power (*P*)	Cutting Speed (*v*)	Feed Rate (*f*)	Depth of Cut (*a*_p_)
*K*_1_	0.7017	0.6033	0.5570	0.5325
*K*_2_	0.5930	0.5693	0.5620	0.5847
*K*_3_	0.5048	0.5343	0.5917	0.5965
*K*_4_	0.5162	0.6090	0.6050	0.6020
*R*	0.1969	0.0747	0.0480	0.0695
Order	laser power > cutting speed > depth of cut > feed rate			

## Data Availability

The datasets used or analyzed during the current study are available from the corresponding author upon reasonable request.
